# First report of V1016G and S989P knockdown resistant (*kdr*) mutations in pyrethroid-resistant Sri Lankan *Aedes aegypti* mosquitoes

**DOI:** 10.1186/s13071-018-3113-0

**Published:** 2018-09-26

**Authors:** Sachini D. Fernando, Menaka Hapugoda, Rushika Perera, Karla Saavedra-Rodriguez, William C. Black, Nissanka K. De Silva

**Affiliations:** 1 Center for Biotechnology, Department of Zoology, Faculty of Applied Sciences, University of Sri Jayewardenepura, Nugegoda, Sri Lanka; 2 Molecular Medicine Unit, Faculty of Medicine, University of Kelaniya, Ragama, Sri Lanka; 3 Department of Microbiology, Immunology and Pathology, Colorado State University, Fort Collins, CO 80523 USA

**Keywords:** *Aedes aegypti*, Sri Lanka, Voltage-gated sodium channel, Pyrethroid resistance, *kdr*

## Abstract

**Background:**

Dengue is a serious arboviral disease in Sri Lanka with a large number of dengue fever (DF) cases every year. Control of the primary vector *Aedes aegypti* depends upon larval habitat source reduction and insecticide application. However, increases in the number of reported cases suggest the inefficiency of current control strategies and the possibility of resistance to currently used insecticides. Early detection of mutations in the voltage-gated sodium channel (*vgsc*) gene that confer knockdown resistance (*kdr*) to pyrethroid insecticides is important in resistance management in vector populations.

**Results:**

Resistance to pyrethroid insecticides was detected in the three populations studied. Polymerase chain reaction was used to detect the presence of two *kdr* mutations F1534C and V1016G. During this process a S989P mutation was also detected in pyrethroid-resistant *Ae. aegypti* populations. These mutations were found to be widespread and frequent in the collections studied.

**Conclusions:**

To our knowledge, this study reveals for the first time the presence of V1016G and S989P mutant alleles in the *vgsc* of Sri Lankan *Ae. aegypti* populations. The spread of the mutant alleles throughout the country poses a threat of increased resistance to pyrethroids. Long-term insecticide applications and indiscriminate use of pyrethroids has led to the evolution of resistance. More strategic and diverse strategies, including novel insecticides with new modes of action and community participation, should be engaged for *Ae. aegypti* control.

**Electronic supplementary material:**

The online version of this article (10.1186/s13071-018-3113-0) contains supplementary material, which is available to authorized users.

## Background

Dengue fever (DF) has been present in Sri Lanka for over four decades and current estimates of the number of DF cases suggest an alarming increase in prevalence progressing towards a hyper-endemic situation. The highest number of cases ever reported in Sri Lanka was in 2017 with 186,101 DF cases. So far, the Sri Lankan health ministry has reported 16,404 DF cases during the first four months of 2018 [1].

Control of dengue infections depends principally on the removal of larval habitats and the use of insecticides [2]. In Sri Lanka, pyrethroid insecticides are most extensively utilized as adulticides by both government authorities and the private sector [3]. These insecticides act quickly as neurotoxins by binding to the gate of the voltage-gated sodium channel (*vgsc*), thus blocking the gate in an open position and thereby preventing development of an electric potential across the membrane. This results in rapid paralysis and death of the mosquito and is often referred to as “knockdown” [4]. Knockdown resistance (*kdr*) is one of the major pyrethroid resistance mechanisms in insects. Genotyping of *kdr* mutant alleles is considered to be a good predictor of the efficacy of pyrethroids and DDT in the field [5]. Eleven mutations have been linked to pyrethroid resistance in *Ae. aegypti.* Mutations G923V, L982W, I1011M and V1016G were first identified in Asian *Ae. aegypti* [6]. Replacements I1011V and V1016I were identified in Latin American countries [7] and the D1763Y mutation was identified in a laboratory reared permethrin-resistant colony [8]**.** Mutations S989P and F1534C have been associated with pyrethroid resistance [9, 10]. T1520I was recently identified in Indian *Ae. aegypti* [11] and mutation V410 has been recently detected [12].

Only five of these mutations have been functionally confirmed to confer pyrethroid resistance. These include S989P, I1011M, V1016G, F1534C [13] and most recently V410L [12]. It has been shown that the F1534C mutation appears first in pyrethroid-treated populations and that survival of the other mutations (e.g. V1016I) ensue [14, 15]. Analysis of *vgsc* mutations in resistant populations around the world has revealed that V1016I is often present in Latin American countries whereas V1016G at the same amino acid residue is found in Southeast Asian countries [13, 16]. Parallel evolution of three mutations, F1534C, V1016I and V410L occur in three different domains IIIS6, IIS6 and IS6, respectively, and has been recently analyzed [17]. This study assumed that either V410L and V1016I might have independently occurred on a C1534 haplotype, or the three mutations independently arose. They suggested that in the presence of pyrethroids, the co-occurrence of V410L and V1016I may provide fitness advantage in the resistant mosquito population [17], thus favoring co-adaptation. Furthermore, co-occurrence of *kdr* mutations has been commonly observed in pyrethroid-resistant populations and has been shown to confer higher levels of resistance.

The present study was undertaken to test for the presence of *vgsc* mutations in three pyrethroid-resistant Sri Lankan *Ae. aegypti* collections. To our knowledge, this study reports the presence of V1016G and S989P mutations in Sri Lanka for the first time.

## Methods

### Mosquito sampling and rearing

Larvae and pupae of *Aedes* mosquitoes were collected in Colombo, Galle and Gampaha districts using ovitraps. One study site in each district was selected based on previous reports of dengue cases and the high frequency of insecticide application. The collected larvae were reared to adults and identified to species using standard taxonomic keys [18]. *Aedes aegypti* adults were artificially blood-fed to generate F_1_ generation eggs. Hatched larvae were fed on fish food and adults on 10% sucrose solution and were reared in an insectary with a temperature of 28 ± 2 °C, relative humidity of 80–100% and a 12:12 h photoperiod. F_1_ mosquitoes were then used for bioassay.

### Susceptibility tests for pyrethroid insecticides

The susceptibility of Sri Lankan *Ae. aegypti* to the insecticides permethrin and deltamethrin was evaluated using the standard WHO protocols [19]. Insecticide papers were purchased from WHO affiliated Universiti Sains Malaysia. From each location, one- to three-day-old non-blood-fed females, maintained on a 10% w/v sucrose solution, were used. An experiment consisted of four replicates of 25 mosquitoes with one control. Test mosquitoes were exposed to 0.05% deltamethrin or 0.75% permethrin impregnated papers for 1 h in the exposure tubes. They were then transferred to holding tubes and provided with a 10% w/v sucrose solution. After 24 h, surviving mosquitoes (resistant) and dead mosquitoes that were incapable of movement (susceptible) were counted.

### DNA extraction and *kdr* mutation genotyping

A total of 281 deltamethrin and permethrin-resistant *Ae. aegypti* that survived insecticide exposure (Table 1) were subjected to DNA extraction using a modified phenol-chloroform method [20]. DNA samples were processed to estimate F1534C and V1016G genotype frequencies.

**Table 1:** Mortality percentages for the pyrethroid insecticides in the *Ae. aegypti* populations

Population	Mortality % (*n* = 100)	
	Permethrin	Deltamethrin
Colombo	10/100	40/100
Gampaha	38/100	44/100
Galle	67/100	80/100

### F1534C AS-PCR genotyping

To detect the F1534C mutation, allele specific PCR (AS-PCR) was performed [21]. PCR was performed in a 25 μl volume with final concentrations of 1.5 mM MgCl_2_, 1× PCR reaction buffer, 0.5 μM forward primer (Phe1534), 0.165 μM forward primer (Cys1534), 0.5 μM common reverse primer (Cpr), 200 μM dNTP mixture, 0.2 U of *Taq* DNA polymerase enzyme and 2 ng of template DNA. Thermal cycling conditions were: initial denaturation at 95 °C for 2 min, followed by 35 cycles of 95 °C for 30 s, 60 °C for 30 s and 72 °C for 30 s, followed by a final extension at 72 °C for 2 min.

### V1016G AS-PCR genotyping

Detection of the V1016G mutation in the *Ae. aegypti vgsc* gene was also carried out following AS-PCR [21]. Each AS-PCR for V1016G was performed in 25 μl reaction volumes. Each reaction consisted of 1.5 mM MgCl_2_, 1× PCR reaction buffer, 0.25 μM forward primer (V1016G), 0.125 μM each reverse primer specific for either glycine (Gly1016R) or valine (Val1016R), 200 μM dNTP mixture, 0.2 U *Taq* DNA polymerase enzyme and 2 ng of template DNA. Thermal cyclic conditions were as: initial denaturation at 94 °C for 2 min, followed by 35 cycles at 94 °C for 30 s, 55 °C for 30 s and 72 °C for 30 s, followed by a final extension at 72 °C for 2 min.

Results for both F1534C and V1016G were visualized by agarose gel electrophoresis. PCR products were loaded onto 4% (TBE) agarose gels and electrophoresis was conducted for 90 min at 65 V with a 50 bp DNA ladder.

### DNA sequencing

To validate the results obtained from the AS-PCR for the V1016G mutation, a fragment of domain II subunit 6 in *vgsc* that contains the V1016G mutation was amplified and sequenced [22]. Each reaction was performed in a 25 μl reaction volume with 1.5 mM MgCl_2_, 1× PCR buffer, 0.5 μM of forward and reverse primers, 200 μM of dNTP mix and 0.4 units of *Taq* DNA polymerase. The PCR thermocycle consisted of 95 °C for 2 min, followed by 35 cycles at 95 °C for 30 s, 63 °C for 30 s and 72 °C for 30 s, followed by a final extension of 72 °C for 2 min. Amplified products were visualized on 1.5% agarose gels (TBE) and were sent to Macrogen (Seoul, Korea) for sequencing.

## Results

Table 1 presents the results for the WHO bioassays. A total of 750 female *Ae. aegypti* mosquitoes collected from three districts were tested for susceptibility to permethrin and deltamethrin adulticides. Mortality rates varied between 10 and 80%. The highest mortality was generated by deltamethrin in all three collections. The lowest mortality was recorded from Colombo district. All three collections exhibited some resistance to pyrethroids. Permethrin resistance was significantly greater in Colombo than in Gampaha (*P* < 0.0001) and the permethrin resistance between Colombo and Galle were also significantly different (*P* < 0.0001). However, the deltamethrin mortality was significantly higher in Galle than in Colombo and Gampaha (*P* < 0.0001) whereas no significant difference was observed between Colombo and Gampaha populations (*P* = 0.6675). Mortality percentages recorded for permethrin and deltamethrin were not significantly different (*P* > 0.05) between the populations (Table 2).

**Table 2:** Two by two contingency analysis of mortality rates provided in Table 1. Probabilities > 0.05 are highlighted in bold

	Colombo: permethrin	Gampaha: permethrin	Galle: permethrin	Colombo: deltamethrin	Gampaha: deltamethrin
Colombo: permethrin					
Gampaha: permethrin	2.20E-16				
Galle: permethrin	2.20E-16	6.61E-05			
Colombo: deltamethrin	1.24E-06	**8.85E-01**	2.10E-04		
Gampaha: deltamethrin	6.79E-08	**4.72E-01**	1.68E-03	**6.68E-01**	
Galle: deltamethrin	2.20E-16	2.01E-09	5.40E-02	1.06E-08	2.34E-07

Table 3 presents the results of genotyping the *vgsc* mutations in the three Sri Lankan collections. Only mosquitoes that survived deltamethrin or permethrin exposures were genotyped (Additional file 1: Table S1). The frequency of the mutation F1534C for permethrin-resistant mosquitoes was highest in the Colombo population (0.64) whereas the frequency of the mutation F1534C for deltamethrin-resistant mosquitoes were highest in Gampaha (0.68). Of the 281 samples genotyped a total of 58 mosquitoes were identified as homozygous (Cys/Cys) in the permethrin-resistant and 42 mosquitoes from deltamethrin-resistant samples. These two populations also carried the G1016 mutation in pyrethroid-resistant *Ae. aegypti*. Sequencing confirmed the presence of V1016G in resistant individuals and interestingly also revealed the presence of the S989P mutation in Colombo and Gampaha collections (Additional file 2: Table S2)*.* No *kdr* resistance alleles in positions 1016 and 989 were detected in either the permethrin or deltamethrin Galle collections.

**Table 3:** AS-PCR genotyping results for the pyrethroid-resistant *Ae. aegypti*

Genotype for resistant mosquito populations	Sample size	Genotype			Resistance allele frequency
F1534C genotype					
Permethrin-resistant		Phe/Phe	Phe/Cys	Cys/Cys	
Colombo	90	13	39	38	0.639
Gampaha	35	5	20	10	0.571
Galle	30	5	15	10	0.583
Deltamethrin-resistant					
Colombo	40	8	18	14	0.575
Gampaha	64	3	35	26	0.680
Galle	22	2	18	2	0.500
V1016G genotype					
Permethrin-resistant		Val/Val	Val/Gly	Gly/Gly	
Colombo	90	88	2	0	0.011
Gampaha	35	32	1	2	0.071
Galle	30	30	0	0	0.000
Deltamethrin-resistant					
Colombo	40	38	0	2	0.050
Gampaha	64	61	1	2	0.039
Galle	22	22	0	0	0.000
S989P genotype					
Permethrin-resistant		Ser/Ser	Ser/Pro	Pro/Pro	
Colombo	40	38	0	2	0.050
Gampaha	64	61	1	2	0.039
Galle	22	22	0	0	0.000
Deltamethrin-resistants					
Colombo	90	88	0	2	0.022
Gampaha	35	32	1	2	0.071
Galle	30	30	0	0	0.000

## Discussion

In Sri Lanka, pyrethroids are extensively and routinely used for vector control and as an emergency measure in the event of an epidemic. Additionally, pyrethroids are readily available for household use and for personal protection. The extensive use of insecticides for vector control initiates the development of insecticide resistance in mosquitoes and adversely affects the efficiency of the vector control programs. The present study documents the presence of V1016G and S989P mutations for the first time in Sri Lanka. It has been proposed that the F1534C mutation arose first in the pyrethroid-treated populations due to its low fitness cost [14, 15]. Without this mutation the V1016G and S989P mutations might have been too deleterious to survive. Thus, the discovery of the F1534C allele in combination with V1016G and S989P in the populations predicts that there is a high likelihood of increases in the frequency of mutations with continuous insecticide pressure which, in turn, predicts higher levels of pyrethroid resistance.

Our analysis of resistance in the three collections indicates a great deal of geographical variation among Sri Lankan cities in both the bioassays (Tables 1, 2) and resistant allele frequencies (Table 3). A recent study in Yaounde, Cameroon, has revealed that both *Ae. aegypti* and *Ae. albopictus* were resistant to 0.05% deltamethrin and have reduced susceptibility to 0.75% permethrin [23]. However, the low level of resistance observed in the study [23] has been attributed to the fact that the discriminating dosages used (permethrin 0.75%) were three times higher than the doses recommended for *Aedes* species (permethrin 0.25%) [24]. Although a high level of resistance was observed in the present study for 0.05% deltamethrin and 0.75% permethrin, this reveals a much higher level of real resistance to recommended dosages at the current study sites. It is very likely that the field samples used in this study were not representative of the overall Sri Lankan population. The sites selected for collection had received large amounts of insecticides, compared to the remaining municipality. This initiative was carried out since the primary objective of the study was to detect novel *kdr* mutations in pyrethroid-resistant populations. Although it would have been interesting to analyze all individuals rather than just the survivors, this was not pursued since we wanted to maximize the chances of detecting mutant alleles.

The F1534C mutation, which is by far the more common mutation that has been recorded in Asian countries, has been detected repeatedly in DDT-/permethrin-resistant mosquito populations. In Thailand, a high frequency of the mutant allele 1534C was discovered, and a country-wide distribution of the allele was observed [22]. In a similar study, the distribution of both V1016G and F1534C mutant alleles has been studied in *Ae. aegypti* populations in Thailand [21]. Their results revealed a significantly positive correlation of the V1016G mutant allele associated with resistance to deltamethrin. However, a study carried out in India revealed a positive correlation between DDT and deltamethrin resistance and the F1534C allele [11]. In Malaysian populations of *Ae. aegypti*, the F1534C mutation has been recorded in association with pyrethroid resistance [25]. Thus, the appearance of the F1534C mutant allele with V1016G and S989P in Sri Lankan *Ae. aegypti* populations is evidence that the gene is under selection by pyrethroids in Sri Lanka. However, co-occurrence of two or three *kdr* mutations has been reported in many countries and is believed to result in a higher level of resistance [13] and thus appears to be the result of long-term application of pyrethroids. Results from Mexico with F1534C and I1016 predict that once the frequency of F1534C begins to increase it will allow other mutations to become established [15]. If the current rate of pyrethroid use continues in Sri Lanka, results from Mexico predict that V1016G will begin to increase rapidly and spread throughout the country, as will the phenotypic resistance.

## Conclusions

In Sri Lanka, as the need to control mosquito population intensifies with the increase in DF cases, the usage of insecticides, particularly pyrethroids, will also intensify. In the light of this, the detection of V1016G and S989P alleles with a high frequency of the F1534C allele predicts rapid selection for pyrethroid-resistant. Thus, it is of utmost importance to detect and monitor the emergence and spread of *kdr* mutations in the country and implement successful resistance management strategies.

## Additional files

Additional file 1: **Table S1.** Genotypes obtained for the individual samples. (DOCX 23 kb)

Additional file 2: **Table S2.** Mutation of F1534C, V1016G and S989P among the *Aedes aegypti* individual samples that were sequenced. Accession numbers are provided for the sequences that are obtained for sequencing domain II subunit 6 of voltage-gated sodium channel gene which consists of the V1016G and S989P mutation. (DOCX 14 kb)

## Abbreviations

DF: Dengue fever
*Kdr*: knockdown resistance
*Vgsc*: voltage gated sodium channel
AS-PCR: allele-specific polymerase chain reaction
