# Supplementary material for: First report of V1016G and S989P knockdown resistant (kdr) mutations in pyrethroid-resistant Sri Lankan Aedes aegypti mosquitoes
Source: Parasit Vectors. 2018 Sep 26;11:526. doi: 10.1186/s13071-018-3113-0 (PMC6158842; doi:10.1186/s13071-018-3113-0)
Supplement: Supplementary file 1 — Table S1. Genotypes obtained for the individual samples. (DOCX 23 kb) [file 13071_2018_3113_MOESM1_ESM.docx]

**Additional file 1: Table S1.** Genotypes obtained for the individual samples.

| No. | Sample ID | Region | Insecticide tested | Genotype | | |
| --- | --- | --- | --- | --- | --- | --- |
|  |  |  |  | F1534C | V1016G | S989P |
| 1 | Gampaha01 | Gampaha | Deltamethrin | FC | VV | SS |
| 2 | Gampaha02 | Gampaha | Deltamethrin | CC | VV | SS |
| 3 | Gampaha03 | Gampaha | Deltamethrin | FF | VV | SS |
| 4 | Gampaha04 | Gampaha | Deltamethrin | FC | VV | SS |
| 5 | Gampaha05 | Gampaha | Deltamethrin | FC | VV | SS |
| 6 | Gampaha06 | Gampaha | Deltamethrin | FC | VV | SS |
| 7 | Gampaha07 | Gampaha | Deltamethrin | FC | VV | SS |
| 8 | Gampaha08 | Gampaha | Deltamethrin | CC | VV | SS |
| 9 | Gampaha09 | Gampaha | Deltamethrin | FC | GG | PP |
| 10 | Gampaha10 | Gampaha | Deltamethrin | FC | VV | SS |
| 11 | Gampaha11 | Gampaha | Deltamethrin | FC | VV | SS |
| 12 | Gampaha12 | Gampaha | Deltamethrin | FF | VV | SS |
| 13 | Gampaha13 | Gampaha | Deltamethrin | CC | VV | SS |
| 14 | Gampaha14 | Gampaha | Deltamethrin | CC | VV | SS |
| 15 | Gampaha15 | Gampaha | Deltamethrin | FF | VV | SS |
| 16 | Gampaha16 | Gampaha | Deltamethrin | FC | VV | SS |
| 17 | Gampaha17 | Gampaha | Deltamethrin | FC | VV | SS |
| 18 | Gampaha18 | Gampaha | Deltamethrin | FC | VV | SS |
| 19 | Gampaha19 | Gampaha | Deltamethrin | FC | VV | SS |
| 20 | Gampaha20 | Gampaha | Deltamethrin | CC | VV | SS |
| 21 | Gampaha21 | Gampaha | Deltamethrin | FC | VG | PP |
| 22 | Gampaha22 | Gampaha | Deltamethrin | FC | VV | SS |
| 23 | Gampaha23 | Gampaha | Deltamethrin | FC | VV | SS |
| 24 | Gampaha24 | Gampaha | Deltamethrin | CC | VV | SS |
| 25 | Gampaha25 | Gampaha | Deltamethrin | FC | GG | SP |
| 26 | Gampaha26 | Gampaha | Deltamethrin | CC | VV | SS |
| 27 | Gampaha27 | Gampaha | Deltamethrin | CC | VV | SS |
| 28 | Gampaha28 | Gampaha | Deltamethrin | CC | VV | SS |
| 29 | Gampaha29 | Gampaha | Deltamethrin | CC | VV | SS |
| 30 | Gampaha30 | Gampaha | Deltamethrin | CC | VV | SS |
| 31 | Gampaha31 | Gampaha | Deltamethrin | CC | VV | SS |
| 32 | Gampaha32 | Gampaha | Deltamethrin | CC | VV | SS |
| 33 | Gampaha33 | Gampaha | Deltamethrin | CC | VV | SS |
| 34 | Gampaha34 | Gampaha | Deltamethrin | FC | VV | SS |
| 35 | Gampaha35 | Gampaha | Deltamethrin | CC | VV | SS |
| 36 | Gampaha36 | Gampaha | Deltamethrin | CC | VV | SS |
| 37 | Gampaha37 | Gampaha | Deltamethrin | FC | VV | SS |
| 38 | Gampaha38 | Gampaha | Deltamethrin | FC | VV | SS |
| 39 | Gampaha39 | Gampaha | Deltamethrin | CC | VV | SS |
| 40 | Gampaha40 | Gampaha | Deltamethrin | FC | VV | SS |
| 41 | Gampaha41 | Gampaha | Deltamethrin | FC | VV | SS |
| 42 | Gampaha42 | Gampaha | Deltamethrin | FC | VV | SS |
| 43 | Gampaha43 | Gampaha | Deltamethrin | CC | VV | SS |
| 44 | Gampaha44 | Gampaha | Deltamethrin | FC | VV | SS |
| 45 | Gampaha45 | Gampaha | Deltamethrin | FC | VV | SS |
| 46 | Gampaha46 | Gampaha | Deltamethrin | FC | VV | SS |
| 47 | Gampaha47 | Gampaha | Deltamethrin | FC | VV | SS |
| 48 | Gampaha48 | Gampaha | Deltamethrin | FC | VV | SS |
| 49 | Gampaha49 | Gampaha | Deltamethrin | CC | VV | SS |
| 50 | Gampaha50 | Gampaha | Deltamethrin | FC | VV | SS |
| 51 | Gampaha51 | Gampaha | Deltamethrin | CC | VV | SS |
| 52 | Gampaha52 | Gampaha | Deltamethrin | FC | VV | SS |
| 53 | Gampaha53 | Gampaha | Deltamethrin | CC | VV | SS |
| 54 | Gampaha54 | Gampaha | Deltamethrin | CC | VV | SS |
| 55 | Gampaha55 | Gampaha | Deltamethrin | FC | VV | SS |
| 56 | Gampaha56 | Gampaha | Deltamethrin | FC | VV | SS |
| 57 | Gampaha57 | Gampaha | Deltamethrin | FC | VV | SS |
| 58 | Gampaha58 | Gampaha | Deltamethrin | CC | VV | SS |
| 59 | Gampaha59 | Gampaha | Deltamethrin | FC | VV | SS |
| 60 | Gampaha60 | Gampaha | Deltamethrin | CC | VV | SS |
| 61 | Gampaha61 | Gampaha | Deltamethrin | CC | VV | SS |
| 62 | Gampaha62 | Gampaha | Deltamethrin | FC | VV | SS |
| 63 | Gampaha63 | Gampaha | Deltamethrin | CC | VV | SS |
| 64 | Gampaha64 | Gampaha | Deltamethrin | FC | VV | SS |
| 65 | Gampaha65 | Gampaha | Permethrin | FF | VV | SS |
| 66 | Gampaha66 | Gampaha | Permethrin | FC | VV | SS |
| 67 | Gampaha67 | Gampaha | Permethrin | FC | VV | SS |
| 68 | Gampaha68 | Gampaha | Permethrin | FC | VV | SS |
| 69 | Gampaha69 | Gampaha | Permethrin | FC | VV | SS |
| 70 | Gampaha70 | Gampaha | Permethrin | FC | GG | PP |
| 71 | Gampaha71 | Gampaha | Permethrin | FC | VV | SS |
| 72 | Gampaha72 | Gampaha | Permethrin | FF | VV | SS |
| 73 | Gampaha73 | Gampaha | Permethrin | FC | VG | PP |
| 74 | Gampaha74 | Gampaha | Permethrin | FF | VV | SS |
| 75 | Gampaha75 | Gampaha | Permethrin | FC | VV | SS |
| 76 | Gampaha76 | Gampaha | Permethrin | FC | VV | SS |
| 77 | Gampaha77 | Gampaha | Permethrin | FC | VV | SS |
| 78 | Gampaha78 | Gampaha | Permethrin | FF | VV | SS |
| 79 | Gampaha79 | Gampaha | Permethrin | FC | VV | SS |
| 80 | Gampaha80 | Gampaha | Permethrin | FF | VV | SS |
| 81 | Gampaha81 | Gampaha | Permethrin | FC | GG | SP |
| 82 | Gampaha82 | Gampaha | Permethrin | FC | VV | SS |
| 83 | Gampaha83 | Gampaha | Permethrin | CC | VV | SS |
| 84 | Gampaha84 | Gampaha | Permethrin | CC | VV | SS |
| 85 | Gampaha85 | Gampaha | Permethrin | CC | VV | SS |
| 86 | Gampaha86 | Gampaha | Permethrin | CC | VV | SS |
| 87 | Gampaha87 | Gampaha | Permethrin | CC | VV | SS |
| 88 | Gampaha88 | Gampaha | Permethrin | CC | VV | SS |
| 89 | Gampaha89 | Gampaha | Permethrin | CC | VV | SS |
| 90 | Gampaha90 | Gampaha | Permethrin | CC | VV | SS |
| 91 | Gampaha91 | Gampaha | Permethrin | CC | VV | SS |
| 92 | Gampaha92 | Gampaha | Permethrin | CC | VV | SS |
| 93 | Gampaha93 | Gampaha | Permethrin | FC | VV | SS |
| 94 | Gampaha94 | Gampaha | Permethrin | FC | VV | SS |
| 95 | Gampaha95 | Gampaha | Permethrin | FC | VV | SS |
| 96 | Gampaha96 | Gampaha | Permethrin | FC | VV | SS |
| 97 | Gampaha97 | Gampaha | Permethrin | FC | VV | SS |
| 98 | Gampaha98 | Gampaha | Permethrin | FC | VV | SS |
| 99 | Gampaha99 | Gampaha | Permethrin | FC | VV | SS |
| 100 | Colombo01 | Colombo | Deltamethrin | FF | VV | SS |
| 101 | Colombo02 | Colombo | Deltamethrin | FC | VV | SS |
| 102 | Colombo03 | Colombo | Deltamethrin | FC | VV | SS |
| 103 | Colombo04 | Colombo | Deltamethrin | FC | VV | SS |
| 104 | Colombo05 | Colombo | Deltamethrin | FC | VV | SS |
| 105 | Colombo06 | Colombo | Deltamethrin | FF | VV | SS |
| 106 | Colombo07 | Colombo | Deltamethrin | FC | GG | PP |
| 107 | Colombo08 | Colombo | Deltamethrin | FC | GG | PP |
| 108 | Colombo09 | Colombo | Deltamethrin | CC | VV | SS |
| 109 | Colombo10 | Colombo | Deltamethrin | FF | VV | SS |
| 110 | Colombo11 | Colombo | Deltamethrin | FF | VV | SS |
| 111 | Colombo12 | Colombo | Deltamethrin | FC | VV | SS |
| 112 | Colombo13 | Colombo | Deltamethrin | FC | VV | SS |
| 113 | Colombo14 | Colombo | Deltamethrin | FF | VV | SS |
| 114 | Colombo15 | Colombo | Deltamethrin | CC | VV | SS |
| 115 | Colombo16 | Colombo | Deltamethrin | FF | VV | SS |
| 116 | Colombo17 | Colombo | Deltamethrin | CC | VV | SS |
| 117 | Colombo18 | Colombo | Deltamethrin | FC | VV | SS |
| 118 | Colombo19 | Colombo | Deltamethrin | FF | VV | SS |
| 119 | Colombo20 | Colombo | Deltamethrin | FC | VV | SS |
| 120 | Colombo21 | Colombo | Deltamethrin | CC | VV | SS |
| 121 | Colombo22 | Colombo | Deltamethrin | FC | VV | SS |
| 122 | Colombo23 | Colombo | Deltamethrin | FF | VV | SS |
| 123 | Colombo24 | Colombo | Deltamethrin | FC | VV | SS |
| 124 | Colombo25 | Colombo | Deltamethrin | FC | VV | SS |
| 125 | Colombo26 | Colombo | Deltamethrin | FC | VV | SS |
| 126 | Colombo27 | Colombo | Deltamethrin | FC | VV | SS |
| 127 | Colombo28 | Colombo | Deltamethrin | FC | VV | SS |
| 128 | Colombo29 | Colombo | Deltamethrin | FC | VV | SS |
| 129 | Colombo30 | Colombo | Deltamethrin | FC | VV | SS |
| 130 | Colombo31 | Colombo | Deltamethrin | CC | VV | SS |
| 131 | Colombo32 | Colombo | Deltamethrin | CC | VV | SS |
| 132 | Colombo33 | Colombo | Deltamethrin | CC | VV | SS |
| 133 | Colombo34 | Colombo | Deltamethrin | CC | VV | SS |
| 134 | Colombo35 | Colombo | Deltamethrin | CC | VV | SS |
| 135 | Colombo36 | Colombo | Deltamethrin | CC | VV | SS |
| 136 | Colombo37 | Colombo | Deltamethrin | CC | VV | SS |
| 137 | Colombo38 | Colombo | Deltamethrin | CC | VV | SS |
| 138 | Colombo39 | Colombo | Deltamethrin | CC | VV | SS |
| 139 | Colombo40 | Colombo | Deltamethrin | CC | VV | SS |
| 140 | Colombo41 | Colombo | Permethrin | FC | VG | PP |
| 141 | Colombo42 | Colombo | Permethrin | CC | VG | PP |
| 142 | Colombo43 | Colombo | Permethrin | FF | VV | SS |
| 143 | Colombo44 | Colombo | Permethrin | CC | VV | SS |
| 144 | Colombo45 | Colombo | Permethrin | CC | VV | SS |
| 145 | Colombo46 | Colombo | Permethrin | FF | VV | SS |
| 146 | Colombo47 | Colombo | Permethrin | CC | VV | SS |
| 147 | Colombo48 | Colombo | Permethrin | CC | VV | SS |
| 148 | Colombo49 | Colombo | Permethrin | CC | VV | SS |
| 149 | Colombo50 | Colombo | Permethrin | CC | VV | SS |
| 150 | Colombo51 | Colombo | Permethrin | FC | VV | SS |
| 151 | Colombo52 | Colombo | Permethrin | CC | VV | SS |
| 152 | Colombo53 | Colombo | Permethrin | CC | VV | SS |
| 153 | Colombo54 | Colombo | Permethrin | CC | VV | SS |
| 154 | Colombo55 | Colombo | Permethrin | FC | VV | SS |
| 155 | Colombo56 | Colombo | Permethrin | FC | VV | SS |
| 156 | Colombo57 | Colombo | Permethrin | FF | VV | SS |
| 157 | Colombo58 | Colombo | Permethrin | FC | VV | SS |
| 158 | Colombo59 | Colombo | Permethrin | FC | VV | SS |
| 159 | Colombo60 | Colombo | Permethrin | FF | VV | SS |
| 160 | Colombo61 | Colombo | Permethrin | FC | VV | SS |
| 161 | Colombo62 | Colombo | Permethrin | FF | VV | SS |
| 162 | Colombo63 | Colombo | Permethrin | FC | VV | SS |
| 163 | Colombo64 | Colombo | Permethrin | FF | VV | SS |
| 164 | Colombo65 | Colombo | Permethrin | FC | VV | SS |
| 165 | Colombo66 | Colombo | Permethrin | FC | VV | SS |
| 166 | Colombo67 | Colombo | Permethrin | FC | VV | SS |
| 167 | Colombo68 | Colombo | Permethrin | FC | VV | SS |
| 168 | Colombo69 | Colombo | Permethrin | FF | VV | SS |
| 169 | Colombo70 | Colombo | Permethrin | FC | VV | SS |
| 170 | Colombo71 | Colombo | Permethrin | FC | VV | SS |
| 171 | Colombo72 | Colombo | Permethrin | FF | VV | SS |
| 172 | Colombo73 | Colombo | Permethrin | CC | VV | SS |
| 173 | Colombo74 | Colombo | Permethrin | CC | VV | SS |
| 174 | Colombo75 | Colombo | Permethrin | CC | VV | SS |
| 175 | Colombo76 | Colombo | Permethrin | FC | VV | SS |
| 176 | Colombo77 | Colombo | Permethrin | CC | VV | SS |
| 177 | Colombo78 | Colombo | Permethrin | CC | VV | SS |
| 178 | Colombo79 | Colombo | Permethrin | CC | VV | SS |
| 179 | Colombo80 | Colombo | Permethrin | FC | VV | SS |
| 180 | Colombo81 | Colombo | Permethrin | CC | VV | SS |
| 181 | Colombo82 | Colombo | Permethrin | CC | VV | SS |
| 182 | Colombo83 | Colombo | Permethrin | FC | VV | SS |
| 183 | Colombo84 | Colombo | Permethrin | FC | VV | SS |
| 184 | Colombo85 | Colombo | Permethrin | FC | VV | SS |
| 185 | Colombo86 | Colombo | Permethrin | FC | VV | SS |
| 186 | Colombo87 | Colombo | Permethrin | CC | VV | SS |
| 187 | Colombo88 | Colombo | Permethrin | CC | VV | SS |
| 188 | Colombo89 | Colombo | Permethrin | CC | VV | SS |
| 189 | Colombo90 | Colombo | Permethrin | FC | VV | SS |
| 190 | Colombo91 | Colombo | Permethrin | FC | VV | SS |
| 191 | Colombo92 | Colombo | Permethrin | FC | VV | SS |
| 192 | Colombo93 | Colombo | Permethrin | FC | VV | SS |
| 193 | Colombo94 | Colombo | Permethrin | FC | VV | SS |
| 194 | Colombo95 | Colombo | Permethrin | FF | VV | SS |
| 195 | Colombo96 | Colombo | Permethrin | FC | VV | SS |
| 196 | Colombo97 | Colombo | Permethrin | FC | VV | SS |
| 197 | Colombo98 | Colombo | Permethrin | CC | VV | SS |
| 198 | Colombo99 | Colombo | Permethrin | FC | VV | SS |
| 199 | Colombo100 | Colombo | Permethrin | FC | VV | SS |
| 200 | Colombo101 | Colombo | Permethrin | CC | VV | SS |
| 201 | Colombo102 | Colombo | Permethrin | CC | VV | SS |
| 202 | Colombo103 | Colombo | Permethrin | CC | VV | SS |
| 203 | Colombo104 | Colombo | Permethrin | CC | VV | SS |
| 204 | Colombo105 | Colombo | Permethrin | CC | VV | SS |
| 205 | Colombo106 | Colombo | Permethrin | CC | VV | SS |
| 206 | Colombo107 | Colombo | Permethrin | CC | VV | SS |
| 207 | Colombo108 | Colombo | Permethrin | CC | VV | SS |
| 208 | Colombo109 | Colombo | Permethrin | CC | VV | SS |
| 209 | Colombo110 | Colombo | Permethrin | CC | VV | SS |
| 210 | Colombo111 | Colombo | Permethrin | CC | VV | SS |
| 211 | Colombo112 | Colombo | Permethrin | FF | VV | SS |
| 212 | Colombo113 | Colombo | Permethrin | FF | VV | SS |
| 213 | Colombo114 | Colombo | Permethrin | FC | VV | SS |
| 214 | Colombo115 | Colombo | Permethrin | FC | VV | SS |
| 215 | Colombo116 | Colombo | Permethrin | FF | VV | SS |
| 216 | Colombo117 | Colombo | Permethrin | FC | VV | SS |
| 217 | Colombo118 | Colombo | Permethrin | FC | VV | SS |
| 218 | Colombo119 | Colombo | Permethrin | FF | VV | SS |
| 219 | Colombo120 | Colombo | Permethrin | FC | VV | SS |
| 220 | Colombo121 | Colombo | Permethrin | FC | VV | SS |
| 221 | Colombo122 | Colombo | Permethrin | CC | VV | SS |
| 222 | Colombo123 | Colombo | Permethrin | FC | VV | SS |
| 223 | Colombo124 | Colombo | Permethrin | CC | VV | SS |
| 224 | Colombo125 | Colombo | Permethrin | FC | VV | SS |
| 225 | Colombo126 | Colombo | Permethrin | FC | VV | SS |
| 226 | Colombo127 | Colombo | Permethrin | CC | VV | SS |
| 227 | Colombo128 | Colombo | Permethrin | CC | VV | SS |
| 228 | Colombo129 | Colombo | Permethrin | FC | VV | SS |
| 229 | Colombo130 | Colombo | Permethrin | CC | VV | SS |
| 230 | Galle01 | Galle | Deltamethrin | FC | VV | SS |
| 231 | Galle02 | Galle | Deltamethrin | FC | VV | SS |
| 232 | Galle03 | Galle | Deltamethrin | FF | VV | SS |
| 233 | Galle04 | Galle | Deltamethrin | FC | VV | SS |
| 234 | Galle05 | Galle | Deltamethrin | FC | VV | SS |
| 235 | Galle06 | Galle | Deltamethrin | FC | VV | SS |
| 236 | Galle07 | Galle | Deltamethrin | FC | VV | SS |
| 237 | Galle08 | Galle | Deltamethrin | FC | VV | SS |
| 238 | Galle09 | Galle | Deltamethrin | FC | VV | SS |
| 239 | Galle10 | Galle | Deltamethrin | FF | VV | SS |
| 240 | Galle11 | Galle | Deltamethrin | FC | VV | SS |
| 241 | Galle12 | Galle | Deltamethrin | CC | VV | SS |
| 242 | Galle13 | Galle | Deltamethrin | FC | VV | SS |
| 243 | Galle14 | Galle | Deltamethrin | FC | VV | SS |
| 244 | Galle15 | Galle | Deltamethrin | CC | VV | SS |
| 245 | Galle16 | Galle | Deltamethrin | FC | VV | SS |
| 246 | Galle17 | Galle | Deltamethrin | FC | VV | SS |
| 247 | Galle18 | Galle | Deltamethrin | FC | VV | SS |
| 248 | Galle19 | Galle | Deltamethrin | FC | VV | SS |
| 249 | Galle20 | Galle | Deltamethrin | FC | VV | SS |
| 250 | Galle21 | Galle | Deltamethrin | FC | VV | SS |
| 251 | Galle22 | Galle | Deltamethrin | FC | VV | SS |
| 252 | Galle23 | Galle | Permethrin | FC | VV | SS |
| 253 | Galle24 | Galle | Permethrin | FC | VV | SS |
| 254 | Galle25 | Galle | Permethrin | CC | VV | SS |
| 255 | Galle26 | Galle | Permethrin | FF | VV | SS |
| 256 | Galle27 | Galle | Permethrin | CC | VV | SS |
| 257 | Galle28 | Galle | Permethrin | FC | VV | SS |
| 258 | Galle29 | Galle | Permethrin | FC | VV | SS |
| 259 | Galle30 | Galle | Permethrin | FC | VV | SS |
| 260 | Galle31 | Galle | Permethrin | CC | VV | SS |
| 261 | Galle32 | Galle | Permethrin | CC | VV | SS |
| 262 | Galle33 | Galle | Permethrin | FF | VV | SS |
| 263 | Galle34 | Galle | Permethrin | FC | VV | SS |
| 264 | Galle35 | Galle | Permethrin | CC | VV | SS |
| 265 | Galle36 | Galle | Permethrin | FC | VV | SS |
| 266 | Galle37 | Galle | Permethrin | FC | VV | SS |
| 267 | Galle38 | Galle | Permethrin | CC | VV | SS |
| 268 | Galle39 | Galle | Permethrin | FF | VV | SS |
| 269 | Galle40 | Galle | Permethrin | FF | VV | SS |
| 270 | Galle41 | Galle | Permethrin | CC | VV | SS |
| 271 | Galle42 | Galle | Permethrin | FC | VV | SS |
| 272 | Galle43 | Galle | Permethrin | FC | VV | SS |
| 273 | Galle44 | Galle | Permethrin | FF | VV | SS |
| 274 | Galle45 | Galle | Permethrin | FC | VV | SS |
| 275 | Galle46 | Galle | Permethrin | FC | VV | SS |
| 276 | Galle47 | Galle | Permethrin | CC | VV | SS |
| 277 | Galle48 | Galle | Permethrin | FC | VV | SS |
| 278 | Galle49 | Galle | Permethrin | CC | VV | SS |
| 279 | Galle50 | Galle | Permethrin | CC | VV | SS |
| 280 | Galle51 | Galle | Permethrin | FC | VV | SS |
| 281 | Galle52 | Galle | Permethrin | FC | VV | SS |
