# Supplementary material for: First report of V1016G and S989P knockdown resistant (kdr) mutations in pyrethroid-resistant Sri Lankan Aedes aegypti mosquitoes
Source: Parasit Vectors. 2018 Sep 26;11:526. doi: 10.1186/s13071-018-3113-0 (PMC6158842; doi:10.1186/s13071-018-3113-0)
Supplement: Supplementary file 2 — Table S2. Mutation of F1534C, V1016G and S989P among the Aedes aegypti individual samples that were sequenced. Accession numbers are provided for the sequences that are obtained for sequencing domain II subunit 6 of voltage-gated sodium channel gene which consists of the V1016G and S989P mutation. (DOCX 14 kb) [file 13071_2018_3113_MOESM2_ESM.docx]

**Additional file 2: Table S2.** Mutation of F1534C, V1016G and S989P among *Aedes aegypti* individual samples that were sequenced. Accession numbers are provided for the sequences that are obtained for sequencing domain II subunit 6 of voltage gated sodium channel gene which consists the V1016G and S989P mutation.

| No. | Sample ID | Region | Insecticide tested | Genotype | | | Accession No. |
| --- | --- | --- | --- | --- | --- | --- | --- |
|  |  |  |  | F1534C | V1016G | S989P |  |
| 1 | Gampaha09 | Gampaha | Deltamethrin | FC | GG | PP | MH645580 |
| 2 | Gampaha21 | Gampaha | Deltamethrin | FC | VG | PP | MH645581 |
| 3 | Gampaha25 | Gampaha | Deltamethrin | FC | GG | SP | MH645582 |
| 4 | Gampaha70 | Gampaha | Permethrin | FC | GG | PP | MH645577 |
| 5 | Gampaha73 | Gampaha | Permethrin | FC | VG | PP | MH645578 |
| 6 | Gampaha81 | Gampaha | Permethrin | FC | GG | SP | MH645579 |
| 7 | Colombo41 | Colombo | Permethrin | FC | VG | PP | MH645575 |
| 8 | Colombo42 | Colombo | Permethrin | CC | VG | PP | MH645576 |
| 9 | Colombo07 | Colombo | Deltamethrin | FC | GG | PP | MH645573 |
| 10 | Colombo08 | Colombo | Deltamethrin | FC | GG | PP | MH645574 |
